# Inter- and Intra-Rater Reliability of a Standardized Infrared Thermographic Cellulite Severity Classification System: A Multi-Rater Study

**DOI:** 10.3390/jcm15187305

**Published:** 2026-09-20

**Authors:** Patrycja Szczepańska-Ciszewska, Wojciech Michał Glinkowski, Andrzej Śliwczyński, Magdalena Głowacka, Anna Erkiert-Polguj, Agata Kmita, Aleksandra Rybak, Barbara Algiert-Zielińska, Maria Koserczyk

**Affiliations:** 1 WSEI University, 4 Projektowa Street, 20-209 Lublin, Poland; andrzej.sliwczynski.ahe@gmail.com (A.Ś.);; 2 Center of Excellence “TeleOrto” for Telediagnostics and Treatment of Disorders and Injuries of the Locomotor System, Department of Medical Informatics and Telemedicine, Medical University of Warsaw, 02-091 Warsaw, Poland; 3 Department of Cosmetology and Aesthetic Dermatology, Faculty of Pharmacy, Medical University of Lodz, 91-151 Lodz, Poland; anna.erkiert-polguj@umed.lodz.pl (A.E.-P.); agata.kmita@umed.lodz.pl (A.K.);

**Keywords:** cellulite, gynoid lipodystrophy, infrared thermography, inter-rater reliability, intra-rater reliability, Gwet’s AC2, thermographic imaging, reproducibility

## Abstract

**Background/Objectives:** Clinical cellulite grading relies on inspection and palpation and is susceptible to examiner variability. A single experienced assessor previously evaluated a five-grade infrared thermographic classification, but its reproducibility across raters was unknown. To assess inter-rater and intra-rater reliability of a standardized thermographic grading workflow applied by trained novice raters. **Methods:** This secondary reliability study included 78 anterior FLIR P620 scans. Five raters analyzed each scan twice, one week apart, using standardized FLIR Tools settings and a ΔT-based 0–IV scale. The prespecified principal agreement analysis used Gwet’s AC2 with quadratic weights across the full 0–IV scale; a sensitivity analysis recalculated AC2 using only the observed categories. Confidence intervals were estimated by scan-level bootstrap. **Results:** The scans yielded 780 ratings, all within grades 0–II. Full five-rater exact agreement was 26.9% and 33.3% in rounds 1 and 2, respectively, while within-one-grade agreement was 89.7% and 92.3%. Observed-category AC2, which restricts quadratic weighting to the grades represented in the data (0–II), was 0.786 (95% CI 0.730–0.840) and 0.845 (0.794–0.885). The corresponding prespecified full-scale AC2 values calculated across the theoretical 0–IV weighting range were 0.949 (0.936–0.962) and 0.963 (0.951–0.972). Intra-rater exact agreement ranged from 66.7% to 97.4%, with 100% agreement within one grade for all raters. **Conclusions:** The workflow demonstrated reproducible ordinal grading within the lower severity categories represented in the dataset, although exact five-rater agreement remained limited. The findings do not establish reliability for grades III–IV, diagnostic accuracy, or clinical utility.

## 1. Introduction

Cellulite, also described as gynoid lipodystrophy, is a common alteration of skin surface topography that most often affects the thighs, buttocks, and hips of postpubertal women [1,2,3]. Its clinical appearance reflects interacting anatomical and physiological processes, including the arrangement of fibrous septa, adipose tissue remodeling, dermal and subcutaneous structural heterogeneity, hormonal influences, and disturbances in local microcirculation [4,5,6,7,8]. Although cellulite is frequently approached as an aesthetic concern, standardized assessment remains relevant to clinical documentation, treatment monitoring, and research. Most established grading approaches depend on inspection, palpation, or photonumeric comparison [2,9,10,11]. These methods are clinically accessible but remain influenced by examiner judgment, posture, muscle contraction, illumination, skin tension, and the selected anatomical field. Validated clinical scales have improved standardization, yet comparisons between instruments and imaging methods continue to show that no single approach fully captures the multidimensional phenotype [9,10,11]. Infrared thermography provides non-contact measurement of skin-surface temperature distribution and may reveal spatial heterogeneity related to perfusion and thermal exchange. In dermatology, infrared thermography has been investigated for severity assessment and follow-up in wound care, skin infection, vascular abnormalities, deep inflammatory disease, and other conditions, but it requires strict attention to acquisition conditions and interpretive learning curves [12]. Within cellulite research, thermography has been used to characterize thermal patterns, support severity staging, and monitor treatment-related changes [13,14,15,16,17]. A five-grade thermographic classification based primarily on the maximum–minimum temperature difference within a predefined region of interest (ROI), supported by descriptive thermal patterns, was previously evaluated against palpation-based assessment [18]. That study addressed preliminary methodological validity but used a single experienced thermographic assessor. Reliability across different raters and stability when the same rater repeats the complete image-analysis workflow are separate measurement properties that require direct evaluation. The present study therefore assessed inter-rater and intra-rater reliability of the standardized thermographic cellulite grading workflow. The specific objective was to quantify the inter- and intra-rater agreement achieved when five trained novice raters independently applied the predefined FLIR Tools settings, ROI placement rule, ΔT calculation, and ordinal classification under controlled offline assessment conditions.

## 2. Materials and Methods

### 2.1. Study Design and Objective

This secondary multi-rater inter- and intra-rater reliability study used previously acquired anonymized thermographic scans from the source methodological validation study [18]. The present reliability study was conceived after publication of the source validation study to address the unresolved reproducibility question arising from its single-assessor design. Thermographic image acquisition had been completed before the present reliability study was designed and conducted. The rater training, two assessment rounds, randomization procedures, and statistical analyses followed a predefined protocol. The thermographic scan was the unit of analysis, and the study did not attempt participant-level inference.

### 2.2. Study Material and Thermographic Acquisition

The final analytical dataset comprised 78 anonymized anterior thermographic scans acquired using a FLIR P620 thermal camera (FLIR P620; Teledyne FLIR LLC, Wilsonville, OR, USA; 640 × 480 pixels; noise-equivalent temperature difference ≤40 mK; spectral range, 7.5–13 μm; emissivity, 0.98). Eligibility required acquisition with the FLIR P620 system specified in the source protocol and complete ratings in both assessment rounds. The technical eligibility criterion was independent of the rater-assigned scores and severity grades. The scans depicted the lower trunk and both thighs in an anterior view. Acquisition was performed with the examined individual standing upright in a relaxed, natural posture, without deliberate contraction of the thigh muscles.

The source imaging protocol was conducted in a controlled environment. A 20-min acclimatization period preceded image acquisition. Recent physical exertion, food intake within 2 h before imaging, and tight clothing were avoided. Environmental and camera-related parameters were standardized as described in the source protocol [18].

### 2.3. Standardized FLIR Tools Analysis and Grading Protocol

The raters received complete anonymized thermographic scans rather than pre-cropped or pre-annotated images. Analysis was performed using FLIR Tools version 6.4.18039.1003 (Teledyne FLIR LLC). In accordance with the instruction manual, all raters used the Rainbow HC palette, a fixed display range of 26.6–36.6 °C, emissivity 0.98, reflected temperature 20.0 °C, measurement distance 1.5 m, ambient temperature 22.2 °C, external optics temperature 20.0 °C, external transmission 1.0, and relative humidity 55.5% [18].

For each scan, the rater independently placed a rectangular ROI measuring 50 × 25 units on the predefined thigh region according to the standardized placement rule. Maximum temperature (Tmax) and minimum temperature (Tmin) were obtained within the ROI, and the temperature difference was calculated as ΔT = Tmax − Tmin. The final grade was determined primarily from the predefined ΔT range: grade 0, 0.5–1.9 °C; grade I, 2.0–2.9 °C; grade II, 3.0–3.9 °C; grade III, 4.0–4.9 °C; and grade IV, 5.0–5.9 °C. Descriptive thermographic patterns served as supportive criteria, whereas the ΔT interval remained the primary classification rule.

**Figure 1:**
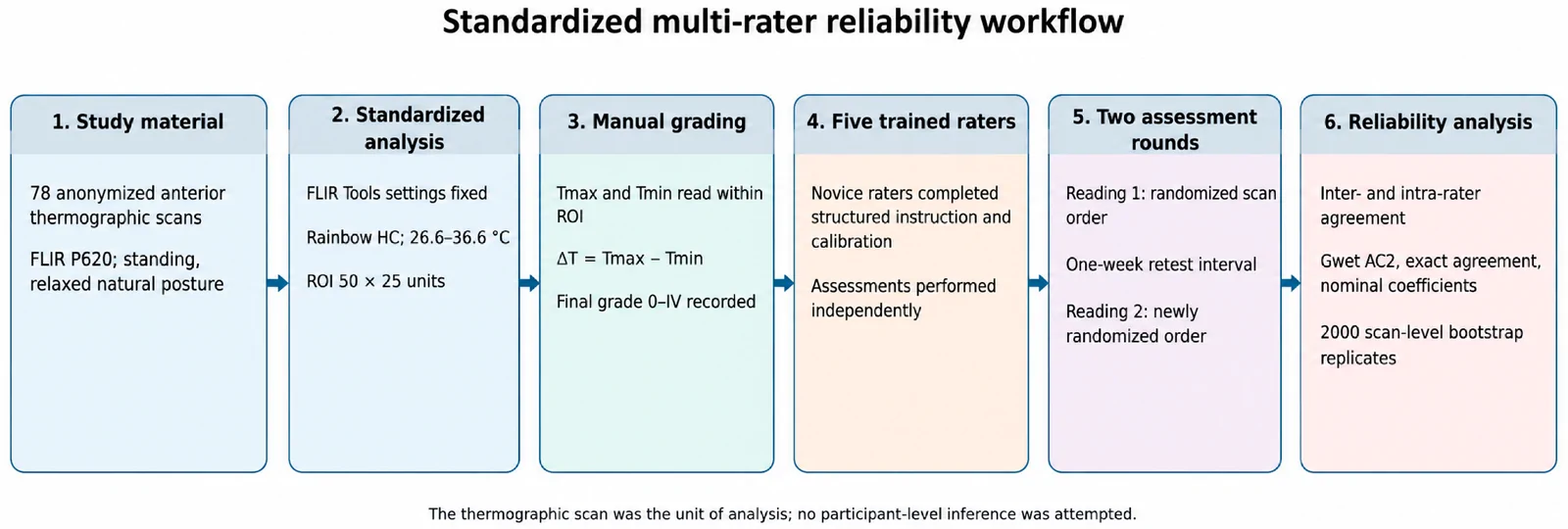
Standardized multi-rater workflow. The thermographic scan was the unit of analysis. Each rater independently completed the full image-analysis and grading sequence in two randomized assessment rounds. This author-designed schematic received artificial-intelligence-assisted graphical layout support; it contains no measured scientific image data.

Only the final ordinal grades were retained in the analytical dataset. ROI coordinates and intermediate Tmax, Tmin, and ΔT values were not archived. Consequently, the present analysis estimates the reliability of the complete end-to-end manual workflow but cannot partition observed disagreement into variability attributable to ROI placement, temperature extraction, ΔT calculation, or application of the grading thresholds. Accordingly, no component-specific reliability estimates were calculated, and no individual source of disagreement was inferred from the final grades. The overall study workflow is summarized in Figure 1.

### 2.4. Raters, Training, Randomization, and Blinding

Five raters with academic training in cosmetology or health sciences participated. None had previous practical experience in infrared thermographic grading of cellulite. Before formal assessment, they completed structured instruction based on the standardized thermographic grading protocol and a supervised calibration session using five representative thermograms covering grades 0–IV.

The calibration thermograms originated from the same source archive. During formal assessment, the complete analytical scan set was presented without grade labels and in randomized order. Because the five calibration thermograms originated from the same source archive, they could reappear unlabeled within the formal analytical set; they did not constitute additional analytical observations. Consequently, visual recognition or anchoring related to prior exposure cannot be excluded.

**Figure 2:**
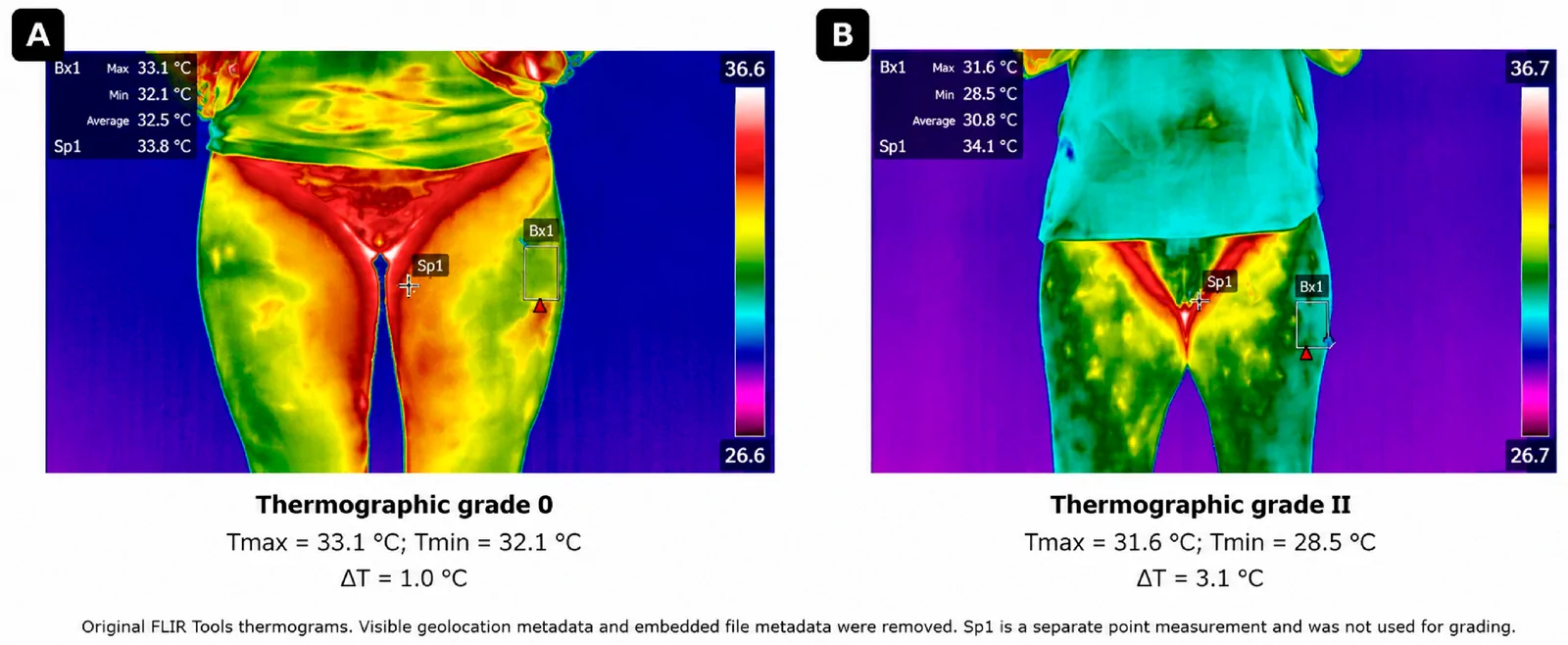
Representative original FLIR Tools analyses from the source image archive after removal of visible geolocation information and embedded file metadata. (**A**) Grade 0: Tmax 33.1 °C, Tmin 32.1 °C, ΔT 1.0 °C. (**B**) Grade II: Tmax 31.6 °C, Tmin 28.5 °C, ΔT 3.1 °C. Sp1 is a separate point-temperature measurement and was not used for grading. Generative artificial intelligence was not used to create or alter the thermographic image content.

Each rater assessed every scan independently. Raters were blinded to the assessments of the other raters and to the clinical palpation grades from the source study. During the second round, they also had no access to their own first-round ratings. The second assessment was performed after a one-week retest interval using a newly randomized scan order. A one-week interval was selected between assessments; however, this duration cannot be assumed to eliminate recognition of previously viewed thermograms. Representative examples of the standardized ROI-based thermographic analysis used for grading are shown in Figure 2.

### 2.5. Statistical Analysis

Inter-rater agreement was calculated separately for the two assessment rounds. Gwet’s AC2 with quadratic weights was selected as the principal ordinal agreement coefficient [19]. Conventional kappa-type coefficients may produce counterintuitive results under high or unequal category prevalence [20,21]. Quadratic weights were defined as:

$$w_{kl}\;=\;1\;-\;{\left(\frac{c_{k}\;-\;c_{l}}{c_{max}\;-\;c_{min}}\right)}^{2}$$

where $w_{kl}$ is the quadratic agreement weight assigned to the pair of categories k and l; $c_{k}$ and $c_{l}$ are the corresponding numerical category scores; and $c_{min}$ and $c_{max}$ are the minimum and maximum scores, respectively, in the category set used to construct the weighting matrix.

The prespecified principal AC2 analysis used the complete predefined category set of grades 0–IV, including grades III and IV, which were not observed in the retained ratings. To evaluate the influence of category-range normalization, AC2 was additionally recalculated using the observed category set of grades 0–II. Accordingly, a one-grade difference received a weight of 0.9375 when the complete 0–IV scale was used and 0.75 when the weighting matrix was defined across the observed 0–II category range.

Because no grades III or IV were observed, the full-scale AC2 should be interpreted as agreement under the weighting structure of the predefined five-grade scale, whereas the observed-category AC2 provides a sensitivity estimate based only on the severity range represented in the analytical data.

Secondary analyses included full five-rater exact agreement, full five-rater within-one-grade agreement, nominal Gwet’s AC1, Krippendorff’s alpha calculated using nominal and interval difference functions [22], and multi-rater Fleiss’ kappa [23].

Intra-rater reliability was assessed for each rater by comparing paired ratings from rounds 1 and 2. The principal intra-rater outputs were exact agreement, agreement within one grade, full-scale AC2, and observed-category sensitivity AC2.

Confidence intervals were estimated with non-parametric scan-level bootstrap resampling using 2000 replicates and percentile 95% intervals. For inter-rater analyses, the complete five-rater rating vector was preserved within each selected scan. For intra-rater analyses, paired round-1 and round-2 ratings were preserved within each rater. The random seed was 20260823. Analyses were performed in Python 3.13.5 using NumPy 2.3.5 and statsmodels 0.14.6. Gwet’s AC1 and AC2 were calculated using author-implemented raw-data functions based on the definitions described by Gwet. Krippendorff’s alpha was calculated using author-implemented disagreement functions with nominal and interval difference metrics. For Fleiss’ kappa, category-count matrices were generated using statsmodels.stats.inter_rater.aggregate_raters, and the coefficient was calculated using statsmodels.stats.inter_rater.fleiss_kappa. The de-identified rating matrix and code reproducing the primary AC2, exact-agreement, within-one-grade, and associated bootstrap analyses are available from the corresponding authors upon reasonable request. Agreement coefficients were interpreted jointly with their confidence intervals, exact agreement, grade distribution, and weighting specification; no universal qualitative cutoff was imposed.

### 2.6. Ethical Considerations

The source validation project received a positive opinion from the Ethics Committee for Scientific Research of the Wyższa Szkoła Biznesu–National Louis University, Nowy Sącz, Poland (Resolution No. 5/2025/2026, dated 10 December 2025). Informed consent had been obtained from all participants in the source study before inclusion of their data in the source dataset. The present investigation was a secondary multi-rater methodological reliability study using previously acquired anonymized thermographic scans and involved no additional participant recruitment, thermographic acquisition, clinical examination, or intervention. All image files prepared for publication were stripped of embedded metadata; visible geolocation information was removed from the representative figures. The study was conducted in accordance with the Declaration of Helsinki.

## 3. Results

### 3.1. Analytical Dataset and Rating Distribution

All five raters completed both assessment rounds for each of the 78 scans, yielding 780 final grades. The predefined scale comprised grades 0–IV; however, the ratings assigned in the analytical dataset covered grades 0–II only, with no grade III or IV ratings recorded. Ratings were strongly concentrated in grades 0 and I, with grade 0 accounting for 66.2% of ratings in round 1 and 73.1% in round 2, whereas grade II accounted for only 4.6% and 3.1%, respectively (Table 1 and Table 2).

Accordingly, empirical reliability estimates for grades III and IV could not be obtained from the present analytical dataset.

### 3.2. Inter-Rater Reliability

Full five-rater exact agreement was 26.9% in round 1 and 33.3% in round 2. The range of all five ratings was within one grade in 89.7% and 92.3% of scans in rounds 1 and 2, respectively. When quadratic weights were defined across the observed categories 0–II, AC2 was 0.786 in round 1 and 0.845 in round 2. The corresponding prespecified full-scale AC2 values, calculated using the complete theoretical 0–IV weighting range, were 0.949 and 0.963, respectively. The difference between these estimates reflects the dependence of quadratic weighting on the category range used for normalization and should be considered when interpreting the magnitude of weighted agreement. Nominal agreement coefficients were lower, ranging from 0.243 to 0.612 across the two assessment rounds (Table 3).

Observed-category AC2 was calculated using grades 0–II represented in the analytical data. Full-scale AC2 used the predefined 0–IV category range; grades III and IV were not observed. The two estimates therefore use different quadratic-weight normalization ranges.

### 3.3. Intra-Rater Reliability

Intra-rater reliability results for the five individual raters are presented in Table 4. Exact agreement between the two rounds ranged from 66.7% to 97.4%. Every rater achieved 100% agreement within one grade. Observed-category sensitivity AC2 ranged from 0.803 to 0.989, with a descriptive mean of 0.923. The corresponding full-scale AC2 values ranged from 0.955 to 0.997, with a descriptive mean of 0.982. Reader 4 showed the lowest exact agreement (66.7%) and observed-category AC2 (0.803), demonstrating heterogeneity in test–retest performance across raters despite the common training protocol. Because process-level measurements were not retained, the source of this rater-specific difference could not be determined.

Observed-category AC2 used the 0–II category range represented in the analytical data; full-scale AC2 used the predefined 0–IV weighting range. Grades III and IV were not observed.

### 3.4. Direction of Changes Between Assessment Rounds

Across the 390 paired scan–rater observations, 325 (83.3%) grades were unchanged, 49 (12.6%) decreased by one grade, and 16 (4.1%) increased by one grade. No paired difference exceeded one grade. Overall, downward changes were more frequent than upward changes (49 vs. 16); however, this pattern was not uniform across raters. Reader 4 accounted for 21 downward and 5 upward changes. Because ROI coordinates and intermediate Tmax, Tmin, and ΔT measurements were not retained, the observed changes cannot be attributed separately to ROI placement, temperature extraction, ΔT calculation, or application of the grading thresholds. No process-level data were available to determine why Reader 4 showed a greater predominance of downward changes than the other raters.

## 4. Discussion

### 4.1. Principal Findings

This study evaluated the reproducibility of an end-to-end manual thermographic grading workflow rather than agreement on precomputed temperatures. Five trained novice raters independently opened each complete scan, applied standardized technical settings, positioned the ROI, extracted temperatures, calculated ΔT, and assigned an ordinal grade. Under these controlled conditions, full five-rater exact agreement was 26.9% and 33.3% across the two rounds, whereas observed-category AC2 was 0.786 and 0.845. The corresponding full-scale AC2 values were higher (0.949 and 0.963), reflecting the different quadratic-weight normalization across the predefined 0–IV category range. All intra-rater disagreements were confined to one adjacent category. Importantly, the analytical dataset represented only grades 0–II, with no ratings in grades III or IV. These findings support reproducibility of the workflow within the lower severity range actually observed but do not justify generalization to the full five-grade scale.

### 4.2. Interpreting Weighted, Exact, and Nominal Agreement

The difference between full-scale AC2, observed-category AC2, and nominal coefficients is central to interpretation. Quadratic weighting assigns partial credit to near agreement and progressively larger penalties to more distant disagreement. Because the weight is normalized by the full category range, a one-grade difference received a weight of 0.9375 when the predefined 0–IV scale was used but 0.75 when weights were recalculated across the observed 0–II range. Accordingly, the observed-category AC2 values of 0.786 and 0.845 provide agreement estimates based on the severity range empirically represented in the analytical data, whereas the higher full-scale AC2 values of 0.949 and 0.963 reflect weighting normalized across the complete predefined 0–IV scale, including unobserved grades III and IV. The full-scale coefficients should therefore not be interpreted as empirical evidence of reliability across grades III–IV. For this reason, interpretation of the present reliability results should prioritize the observed-category sensitivity estimates together with exact and within-one-grade agreement, while retaining the full-scale AC2 as the prespecified analysis under the original five-grade weighting structure.

The lower nominal AC1, Fleiss kappa, and Krippendorff alpha values are not statistical contradictions. Nominal measures treat a disagreement between grades 0 and I in the same way as a disagreement between grades 0 and IV and are affected by marginal prevalence. Conversely, a high weighted coefficient should not be presented in isolation as proof of practical interchangeability. Full five-rater exact agreement remained 26.9–33.3%, showing that complete category identity across all raters was uncommon. Reporting both weighted and exact indices therefore provides a more balanced account of performance. The clinical meaning of adjacent grades also remains uncertain. The observed disagreements were predominantly between adjacent categories, consistent with classification uncertainty near predefined ordinal thresholds. However, the present study did not determine whether a one-grade difference changes prognosis, treatment choice, or patient-relevant outcomes. Adjacent disagreement should therefore be described as ordinally close rather than automatically as clinically unimportant.

### 4.3. Standardization, Training, and Rater Heterogeneity

The protocol standardized variables that can materially influence thermographic appearance: camera system, anterior view, posture, acclimatization, palette, temperature range, emissivity, environmental parameters, ROI dimensions, and calculation rule. Such standardization was intended to reduce technical variation and give all raters a consistent framework for image analysis and grading. The results nevertheless do not show that prior experience is irrelevant. Reader 4 had the lowest exact test–retest agreement (66.7%) and observed-category AC2 (0.803) and accounted for 21 downward and 5 upward changes between assessment rounds. Because process-level measurements and behavioral data were not retained, the source of this rater-specific pattern cannot be determined. Potential explanations involving ROI placement, temperature extraction, threshold application, recognition, attention, or order effects would therefore be speculative.

The five calibration thermograms were drawn from the same source archive and could reappear without labels in the formal randomized set. Although these images represented only a small fraction of the analytical set and did not constitute additional observations, prior exposure may have introduced recognition or anchoring effects. Accordingly, interpret the intra-rater reliability estimates with the possibility of upward bias from image recognition or anchoring. Future reliability studies should use an external calibration set or prospectively reserve training images to exclude from formal scoring. A longer retest interval and a larger image pool may also reduce recognition-related inflation of intra-rater agreement.

### 4.4. Dermatologic and Biological Context

The broader dermatology literature supports infrared thermography as a potentially useful adjunct for documenting temperature distribution in selected inflammatory, infectious, vascular, and wound-related conditions, while emphasizing the influence of protocol quality, disease context, and operator training [12]. These observations support the methodological rationale for standardized thermal imaging but do not establish that a temperature pattern is disease-specific.

Cellulite is multifactorial, and local thermal heterogeneity may reflect a combination of microcirculatory variation, tissue architecture, adipose remodeling, and inflammatory processes. Future studies could combine standardized thermography with clinical grading, patient-reported measures, microvascular assessment, and selected biological markers to determine whether reproducibly classified thermal patterns have mechanistic, clinical, or prognostic significance.

### 4.5. Current Clinical Scope

At present, the method is best positioned as a standardized research and documentation workflow. Potential research applications include harmonized image annotation, standardized documentation of thermographic scans acquired under consistent conditions, rater training, and development of automated or semi-automated thermographic analysis. The present study did not evaluate diagnostic accuracy, responsiveness to clinical change, treatment monitoring, patient-reported outcomes, or decision utility. Any potential clinical use of thermography would currently be adjunctive to, rather than a replacement for, history, inspection, palpation, and established clinical scales. The current results do not support its use as a stand-alone diagnostic test, a replacement for physical examination, or a basis for treatment selection.

### 4.6. Strengths, Limitations, and Future Research

Strengths include the use of a predefined five-grade classification system, standardized camera and software parameters, independent end-to-end analysis by five raters, repeated scoring in a newly randomized order, explicit reporting of exact and weighted agreement, sensitivity analysis addressing the category-range dependence of quadratic weights, and scan-level bootstrap confidence intervals.

Several limitations require emphasis. First, the analysis was scan-based and used a previously acquired single-center archive; no participant-level inference was attempted, and the reliability model did not incorporate participant-level linkage. Because participant-level linkage was unavailable, potential within-participant dependence among scans could not be modeled. The scan-level bootstrap confidence intervals should therefore be interpreted with this limitation in mind. Second, the rating distribution was markedly imbalanced and included only grades 0–II. Reliability of grades III and IV could not be estimated empirically. Moreover, the higher full-scale AC2 values were materially influenced by normalization of quadratic weights across the complete predefined 0–IV category range, including the unobserved grades III and IV. Accordingly, the observed-category AC2 estimates provide the more conservative description of weighted agreement within the severity range represented in the analytical data. Third, all raters were trained novices; performance cannot be assumed to represent experienced clinicians, thermographers, or untrained users. Fourth, the one-week retest interval may have permitted recognition of previously viewed scans, and the five calibration thermograms originated from the same source archive and could reappear unlabeled in the formal assessment set. Recognition or anchoring related to prior exposure therefore cannot be excluded and may have biased the intra-rater agreement estimates upward. Fifth, only final ordinal grades were archived; ROI coordinates and intermediate Tmax, Tmin, and ΔT measurements were not retained. Consequently, disagreement could not be partitioned into variability attributable to ROI placement, temperature extraction, ΔT calculation, or application of the grading thresholds. Sixth, the controlled offline task did not reproduce live clinical acquisition or heterogeneous real-world conditions. Finally, the study did not assess diagnostic accuracy, concurrent validity against a reference standard, responsiveness, treatment outcomes, or decision utility.

Future work should prospectively recruit a severity-balanced cohort with adequate representation of grades III and IV, retain participant-level linkage, use a separate training archive, and store ROI coordinates and all intermediate temperature measurements. Comparative reliability should be examined among novice raters, experienced thermographers, and clinicians. Multicenter studies should assess different devices and acquisition environments. Automated ROI placement should be investigated as a potential means of reducing operator-related variability. Clinical validation should then address agreement with established cellulite scales, responsiveness to longitudinal change, patient-reported outcomes, and whether any thermographic information improves decisions beyond routine examination [9,10,12,18,24].

## 5. Conclusions

Within the lower severity categories represented in the analytical dataset, a standardized FLIR Tools workflow produced reproducible ordinal grading among five trained novice raters under controlled offline conditions. Full five-rater exact agreement was 26.9–33.3%, whereas observed-category AC2 was 0.786–0.845; the higher full-scale AC2 estimates depended materially on normalization of quadratic weights across the complete theoretical 0–IV scale. Because no grade III or IV ratings were observed, reliability across the full predefined five-grade scale remains unestablished. The evaluated thermographic workflow should currently be regarded as a standardized image-based approach for documentation and research, not as a diagnostic substitute for clinical examination or an independently validated tool for clinical decision-making.

## Figures and Tables

**Table 1:** Characteristics of the analytical rating dataset.

Characteristic	Value
Analytical structure	78 scans; 5 raters; 2 rounds; 780 ratings
Camera, view, and posture	FLIR P620; anterior; upright relaxed standing position
Unit of analysis	Thermographic scan
Predefined ordinal scale	0–IV
Observed grades	0–II; no grade III or IV assignments

**Table 2:** Distribution of grades across assessment rounds.

Grade	Round 1, *n* (%)	Round 2, *n* (%)
0	258 (66.2%)	285 (73.1%)
I	114 (29.2%)	93 (23.8%)
II	18 (4.6%)	12 (3.1%)
III	0 (0.0%)	0 (0.0%)
IV	0 (0.0%)	0 (0.0%)
Total	390 (100.0%)	390 (100.0%)

**Table 3:** Inter-rater agreement in the two assessment rounds.

Statistic	Round 1	95% CI	Round 2	95% CI
Full five-rater exact agreement	26.9%	16.7–38.5%	33.3%	23.1–43.6%
Full five-rater within-one-grade agreement	89.7%	83.3–96.2%	92.3%	85.9–97.4%
Gwet AC2, quadratic, observed grades 0–II	0.786	0.730–0.840	0.845	0.794–0.885
Gwet AC2, quadratic, full scale 0–IV	0.949	0.936–0.962	0.963	0.951–0.972
Gwet AC1, nominal	0.538	0.462–0.616	0.612	0.536–0.687
Fleiss kappa, nominal	0.257	0.130–0.381	0.243	0.114–0.357
Krippendorff alpha, nominal	0.259	0.132–0.383	0.245	0.116–0.359
Krippendorff alpha, interval	0.320	0.135–0.485	0.327	0.147–0.482

**Table 4:** Intra-rater agreement between assessment rounds.

Rater	Exact Agreement (95% CI)	Within One Grade	AC2 Observed 0–II (95% CI)	AC2 Full 0–IV (95% CI)
Reader 1	97.4% (93.6–100.0%)	100.0%	0.989 (0.969–1.000)	0.997 (0.993–1.000)
Reader 2	88.5% (80.8–94.9%)	100.0%	0.960 (0.927–0.984)	0.990 (0.982–0.996)
Reader 3	79.5% (70.5–88.5%)	100.0%	0.919 (0.874–0.957)	0.980 (0.970–0.989)
Reader 4	66.7% (56.4–76.9%)	100.0%	0.803 (0.736–0.871)	0.955 (0.939–0.970)
Reader 5	84.6% (75.6–92.3%)	100.0%	0.946 (0.910–0.976)	0.987 (0.978–0.994)

## Data Availability

The de-identified scan-level rating matrix and the code reproducing the primary AC2, exact-agreement, within-one-grade, and associated bootstrap analyses are available from the corresponding authors upon reasonable request. Detailed secondary agreement outputs are provided in Supplementary File S2. Raw thermographic images are not publicly available because of privacy, source-consent, and data-governance restrictions.

## Supplementary Materials

The following supporting information can be downloaded at: https://www.mdpi.com/article/10.3390/jcm15187305/s1, Supplementary File S1: Standardized FLIR Tools rating instruction and translated classification atlas. Supplementary File S2: Detailed inter-rater and intra-rater outputs, weighting specification, bootstrap procedures, and interpretation boundaries.
