# Supplementary material for: Inter- and Intra-Rater Reliability of a Standardized Infrared Thermographic Cellulite Severity Classification System: A Multi-Rater Study
Source: J Clin Med. 2026 Sep 20;15(18):7305. doi: 10.3390/jcm15187305 (PMC13608519; doi:10.3390/jcm15187305)
Supplement: Supplementary file 1 [file jcm-15-07305-s001.zip › jcm-4480954-supplementary File S1.pdf]

Supplementary File S1. Standardized Thermographic Rating Manual and Cellulite Severity Classification Atlas.

This supplementary file provides the complete reader instruction manual used for standardized thermographic image assessment. It includes the FLIR Tools software settings, ROI placement instructions, temperature scale parameters, ΔT-based grading thresholds, and descriptive thermographic criteria for cellulite grades 0–IV. The file also includes representative examples and pattern descriptors used during reader training and formal image evaluation. This material is provided to support the reproducibility of the grading protocol and facilitate independent replication of the thermographic cellulite severity classification procedure.

S1.1 Purpose of the Manual

This manual was developed to standardize infrared thermographic image assessment and cellulite severity grading across independent raters participating in the reliability study. The document provides detailed instructions for image visualization, software settings, region-of-interest placement, temperature measurement, interpretation of thermographic patterns, and assignment of cellulite severity grades according to the predefined classification system.

The manual was used during rater training and formal image evaluation to ensure methodological consistency and minimize observer-related variability. Its purpose is to facilitate reproducible application of the thermographic cellulite severity classification system and to support independent replication of the assessment protocol in future research studies.

S1.2 Required Software

Thermographic image assessment was performed using FLIR Tools software, version 6.4.18039.1003 (Teledyne FLIR LLC). The software was used to visualize thermographic images, apply standardized display settings, define the region of interest, and extract the maximum and minimum temperatures within the selected area.

All raters were instructed to use the same software version and the same predefined image visualization parameters throughout the assessment process. No automated image classification algorithm was used. Final cellulite severity grading was performed manually by each rater according to the standardized thermographic classification criteria provided in this manual.

S1.3 Standardized Image Settings

Before grading, all thermographic images were displayed using the same predefined visualization and measurement settings. The use of fixed settings was required to ensure that all raters interpreted images under identical technical conditions.

The following standardized settings were used for all assessments:

Parameter	Setting
Software	FLIR Tools v6.4.18039.1003
Palette	Rainbow HC
Thermal scale range	26.6–36.6 °C
Emissivity	0.98
Reflected temperature	20.0 °C
Measurement distance	1.5 m
Ambient temperature	22.2 °C
External optics temperature	20.0 °C
External transmission	1.0
Relative humidity	55.5%

Raters were instructed not to modify the palette, temperature scale, emissivity, distance, or environmental parameters during the assessment. Any deviation from these settings could alter the visual appearance of the thermographic pattern and affect the grading of cellulite severity.

S1.4 Region of Interest (ROI) Placement Procedure

A standardized rectangular region of interest (ROI) measuring 50 × 25 units was used for all thermographic assessments. Consistent ROI placement was considered essential to minimize measurement variability and ensure comparability between raters.

The ROI was positioned on the left thigh region of each thermographic image according to the predefined acquisition protocol. During image assessment, raters were instructed to:

1. Open the thermographic image in FLIR Tools software.
2. Verify that all standardized image settings described in Section S1.3 were correctly applied.
3. Select a rectangular ROI measuring 50 × 25 units.
4. Position the ROI on the predefined left thigh area according to the reference example.
5. Ensure that the ROI remained entirely within the target anatomical region and did not extend beyond the skin surface boundaries.
6. Avoid subjective repositioning of the ROI to emphasize warmer or cooler regions.

After ROI placement, the software automatically generated the maximum temperature (Tmax), minimum temperature (Tmin), and mean temperature values within the selected area. These measurements were subsequently used to classify the severity of cellulite.

S1.5 Temperature Difference Calculation

Cellulite severity grading was based primarily on the temperature difference between the maximum and minimum temperature values recorded within the predefined ROI.

The temperature difference was calculated as:

$\Delta T = T_{max} - T_{min}$

where:

- T_{max} represents the maximum temperature measured within the ROI;
- T_{min} represents the minimum temperature measured within the ROI;
- ΔT represents the temperature difference used for thermographic cellulite severity grading.

After placing the ROI, raters recorded T_{max} and T_{min} values displayed by FLIR Tools software. The ΔT value was then calculated for each image and used as the primary quantitative criterion for assigning the cellulite severity grade.

The ΔT value was interpreted alongside the thermographic pattern and histogram morphology. In borderline cases, raters were instructed to consider both the numerical ΔT range and the corresponding visual thermographic descriptors provided in the classification table.

S1.6 Table S1. Thermographic Cellulite Severity Classification System

Grade	ΔT
0	0.5–1.9°C
I	2.0–2.9°C
II	3.0–3.9°C
III	4.0–4.9°C
IV	5.0–5.9°C

S1.7 Thermographic Cellulite Severity Classification System

Cellulite severity was classified using a predefined five-grade ordinal thermographic scale ranging from grade 0 to grade IV. The classification was based on the ΔT value calculated within the predefined ROI and supported by characteristic thermographic pattern descriptors.

Table S1. Thermographic Cellulite Severity Classification System

Grade	ΔT Range	Thermographic Interpretation
0	0.5–1.9 °C	Uniform thermal distribution with no visible thermographic heterogeneity
I	2.0–2.9 °C	Mild thermal irregularity with small yellow-green dots or spots on a predominantly blue-green background
II	3.0–3.9 °C	Moderate thermal heterogeneity with more visible green-yellow areas and small dot-like irregularities
III	4.0–4.9 °C	Marked thermal heterogeneity with yellow-red patches and a “leopard skin” pattern
IV	5.0–5.9 °C	Severe thermal heterogeneity with intense contrast, red-yellow dominance, and low-temperature void-like areas described as “black holes.”

The final grade was assigned by matching the calculated ΔT value with the corresponding grade range and verifying consistency with the visual thermographic pattern. In cases where the numerical ΔT value and pattern appearance were not fully concordant, raters were instructed to prioritize the predefined ΔT range while using the pattern descriptors as supportive criteria.

S1.7 Classification Atlas

The classification atlas provides representative examples of the thermographic appearance corresponding to each cellulite severity grade. For each grade, the atlas should include a representative thermogram, the corresponding histogram or temperature distribution pattern, and a short descriptive interpretation. The examples are intended to support standardized visual interpretation during rater training and image evaluation.

Grade 0

Representative thermographic example (Figure S1).

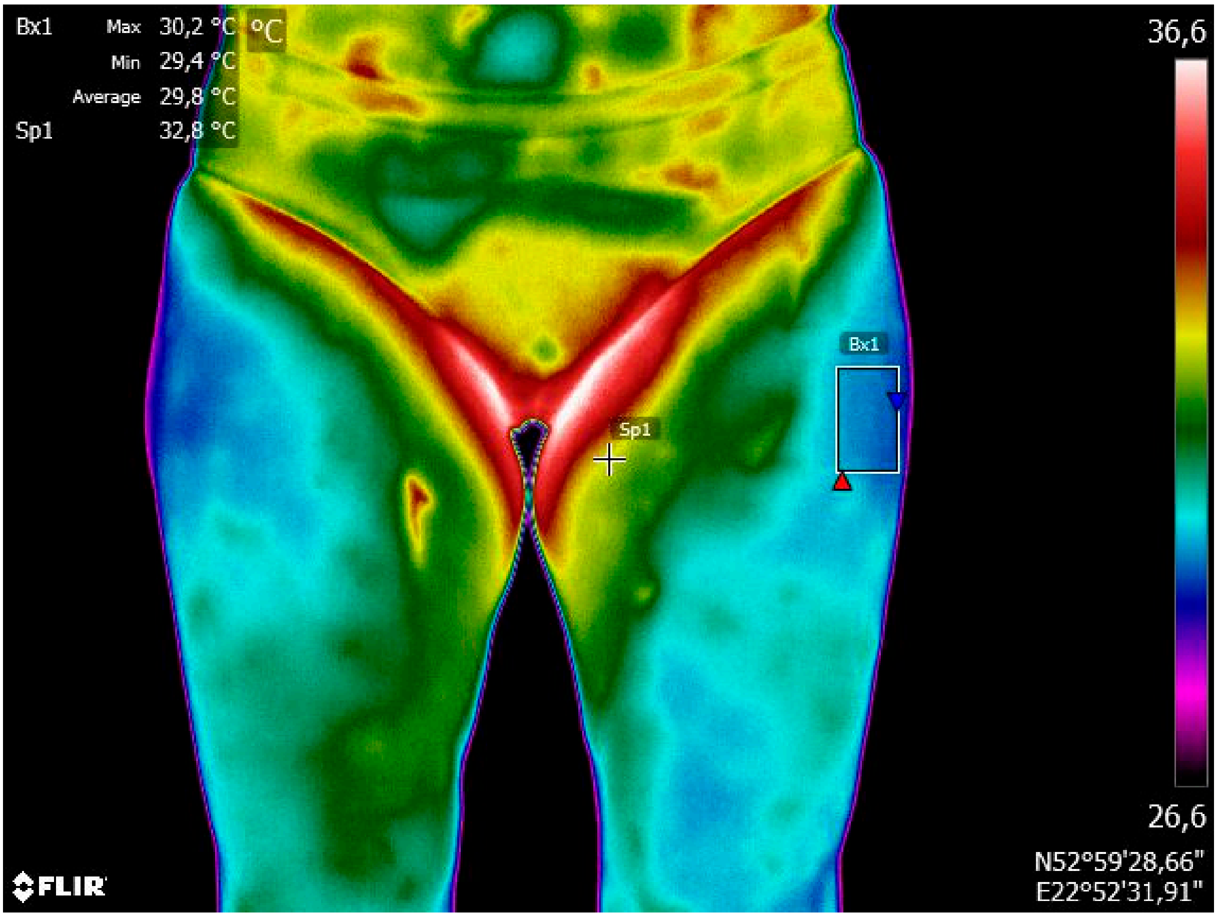

Description: Grade 0 corresponds to a ΔT of 0.5–1.9 °C. The thermographic image shows uniform skin warming with no relevant thermal heterogeneity. The color pattern is predominantly homogeneous, typically in shades of blue and green, without visible dots, patches, mottling, or sharply demarcated areas.

Grade I

Representative thermographic example (Figure S2).

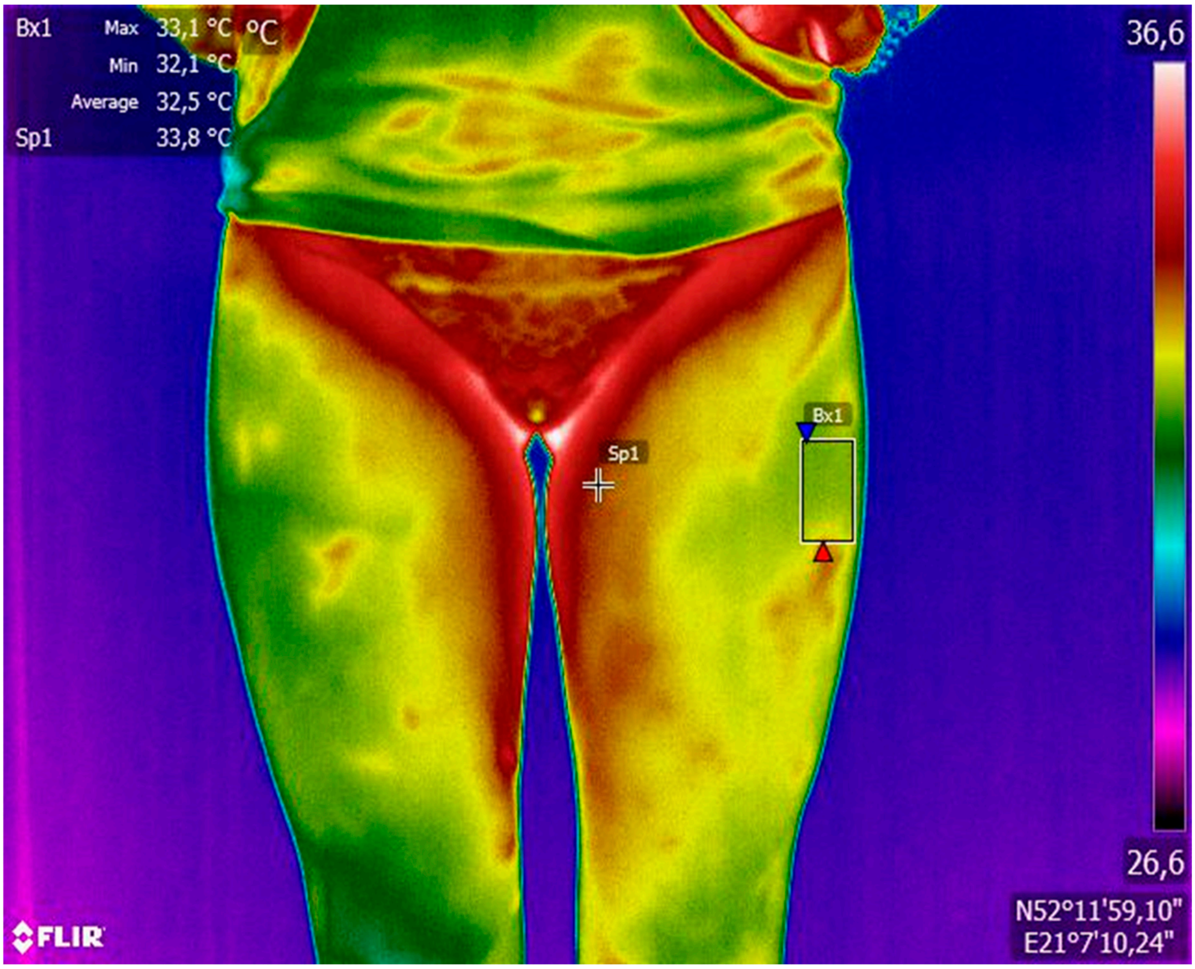

Description: Grade I corresponds to a ΔT of 2.0–2.9 °C. The thermographic image shows mild thermal heterogeneity with small yellow-green dots, spots, or discrete areas on a predominantly blue-green background. Changes are limited and clearly demarcated.

Grade II

Representative thermographic example (Figure S3).

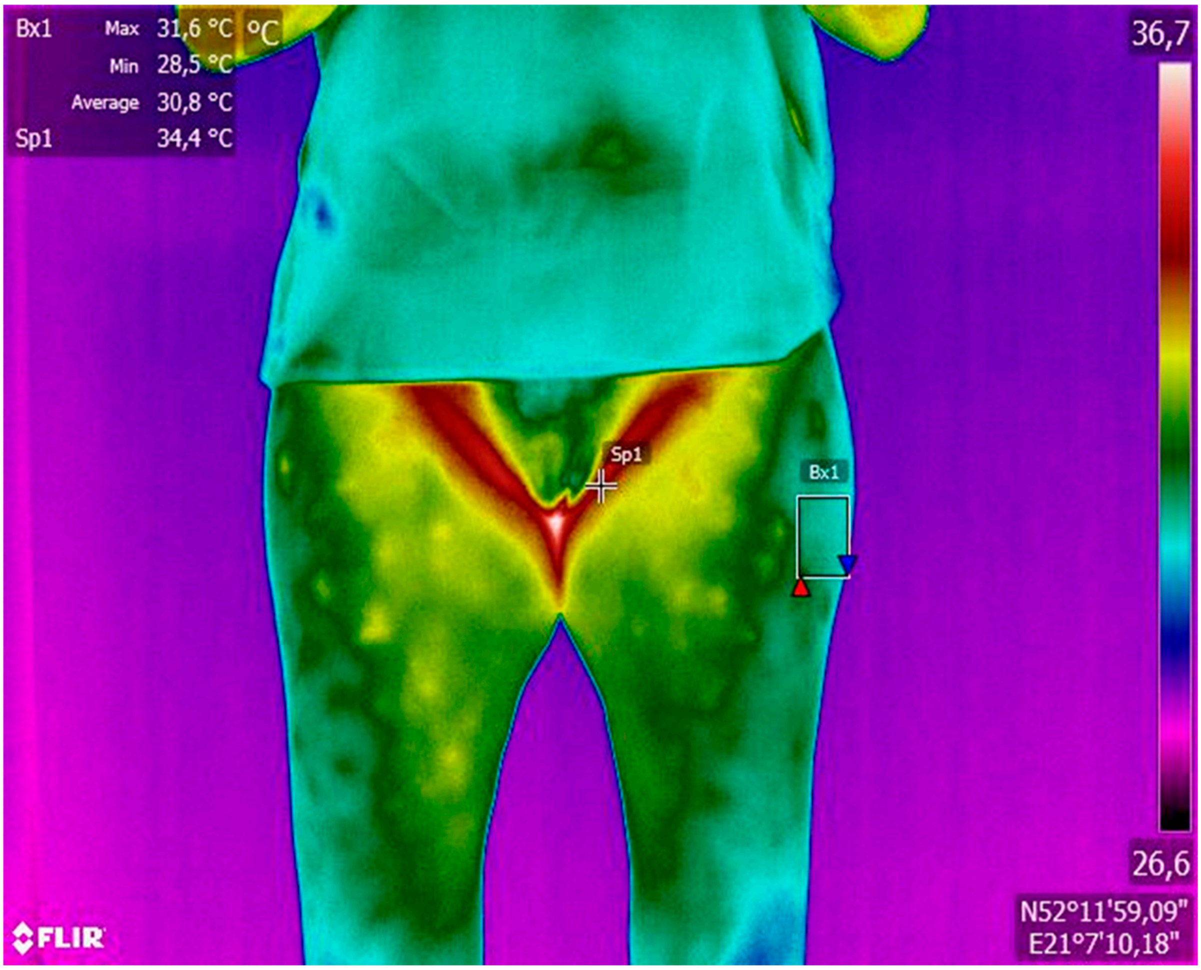

Description: Grade II corresponds to a ΔT of 3.0–3.9 °C. The thermographic image shows more visible thermal heterogeneity, with green-yellow areas and small dot-like irregularities on a blue-green background. The pattern is more pronounced than in grade I but does not yet show extensive red-yellow patching.

Grade III

Representative thermographic example (Figure S4).

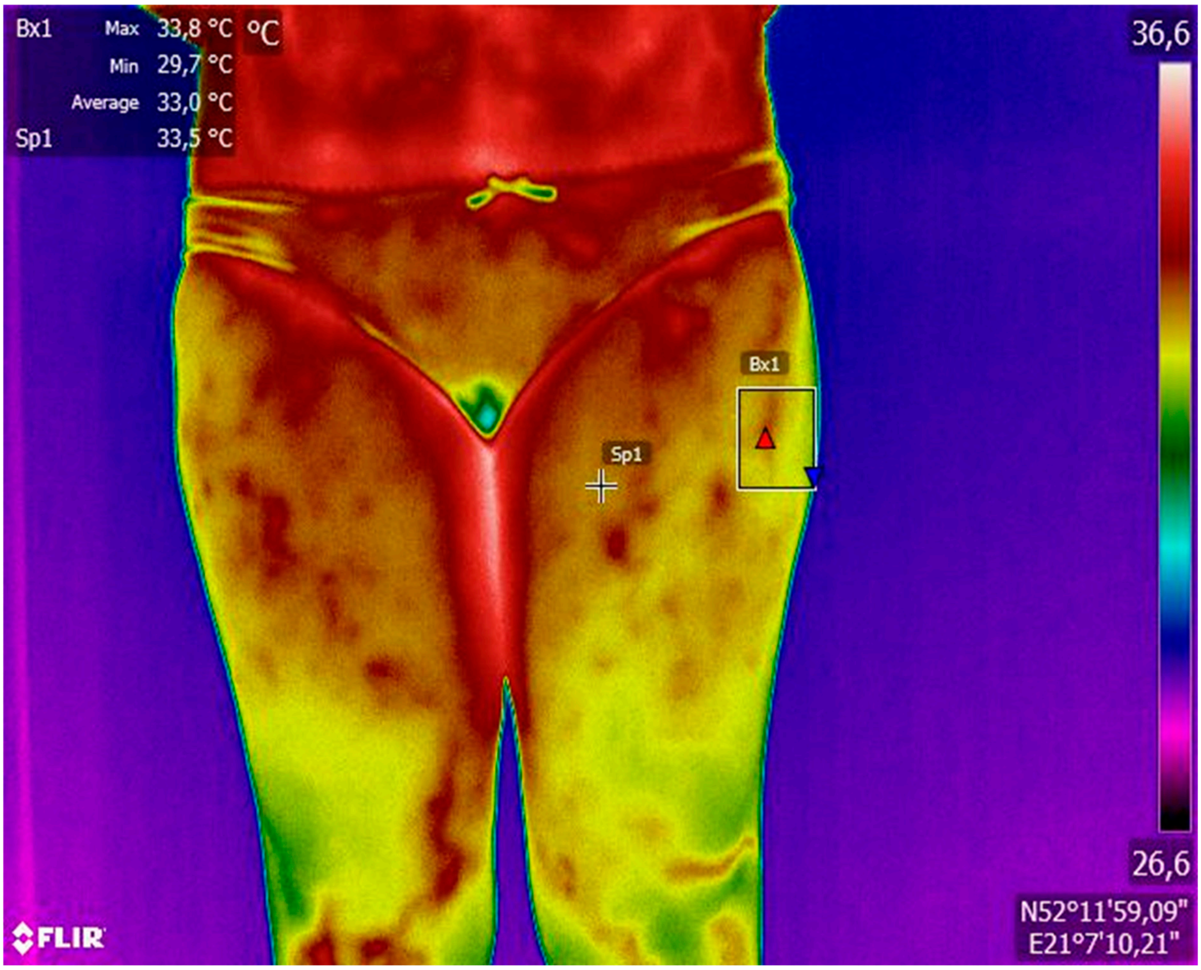

Description: Grade III corresponds to a ΔT of 4.0–4.9 °C. The thermographic image shows marked thermal heterogeneity with yellow-red patches, visible mottling, and a characteristic "leopard skin" pattern. Lesions are more diffuse and clearly visible, with stronger contrast between warmer and cooler areas.

Grade IV

Representative thermographic example (Figure S5).

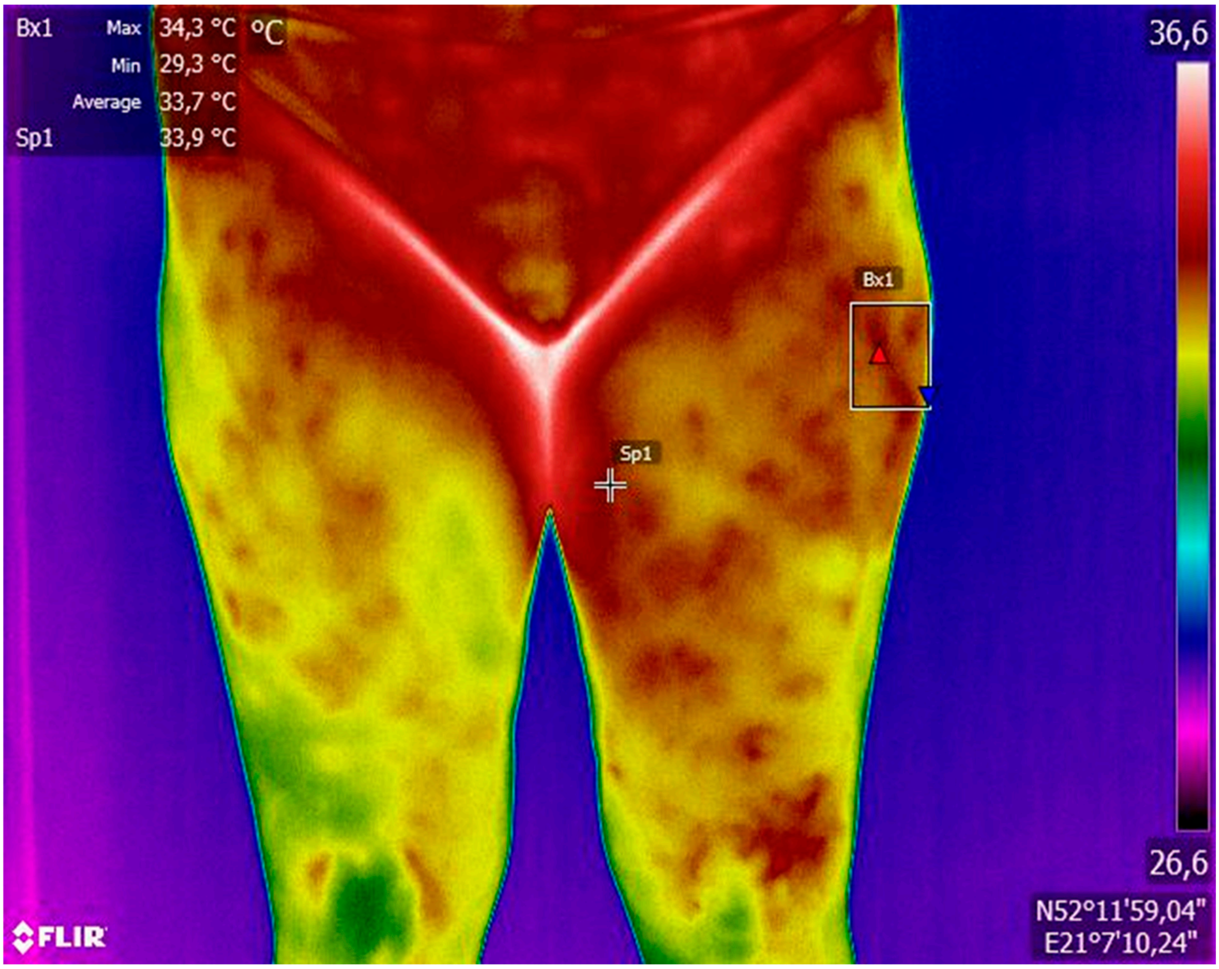

Description: Grade IV corresponds to a ΔT of 5.0–5.9 °C. The thermographic image shows severe thermal heterogeneity with intense red-yellow dominance, high contrast, and low-temperature void-like areas described as “black holes.” The pattern is markedly irregular, with clearly demarcated patches, mottling, and pronounced thermal contrast.

S1.8 Reader Instructions

Raters were instructed to follow the same standardized sequence for every thermographic image. The purpose of this procedure was to ensure consistency of image interpretation and to minimize variability related to software settings, ROI placement, and grade assignment.

For each image, raters completed the following steps:

1. Open the thermographic image in FLIR Tools software.
2. Verify that the standardized image settings are correctly applied.
3. Place the predefined rectangular ROI on the target thigh region.
4. Record the maximum temperature value within the ROI.
5. Record the minimum temperature value within the ROI.
6. Calculate the temperature difference using the formula $\Delta T = T_{max} - T_{min}$.
7. Assign the cellulite severity grade according to the predefined ΔT range.
8. Verify whether the thermographic pattern is consistent with the assigned grade.
9. Record the final score in the rating sheet.

Raters were instructed to avoid changing the image palette, temperature scale, emissivity, distance, or environmental settings during the assessment. In borderline cases, raters were instructed to follow the ΔT-based threshold as the primary criterion and use thermographic pattern descriptors as supportive information.

S1.9 Examples of Borderline Cases

Borderline cases were defined as thermographic images in which the ΔT value was close to the threshold between two adjacent severity categories, or in which the visual thermographic pattern showed features overlapping between neighboring grades.

A common example was the borderline between grade I and grade II. Grade I corresponds to ΔT values of 2.0–2.9 °C and is characterized by mild thermal irregularity with small yellow-green dots or discrete spots. Grade II corresponds to ΔT values of 3.0–3.9 °C and shows more pronounced thermal heterogeneity, with more visible green-yellow areas and dot-like irregularities.

In cases close to the threshold between grade I and grade II, raters were instructed to prioritize the calculated ΔT value. Thermographic pattern appearance and histogram morphology were used as supportive criteria to confirm the final grade. If the ΔT value clearly fell within one predefined range, the corresponding grade was assigned even if some visual features overlapped with the neighboring category.

This rule was applied consistently across all adjacent-grade borderline cases. The purpose was to reduce reliance on subjective visual interpretation and improve the reproducibility of the grading procedure.

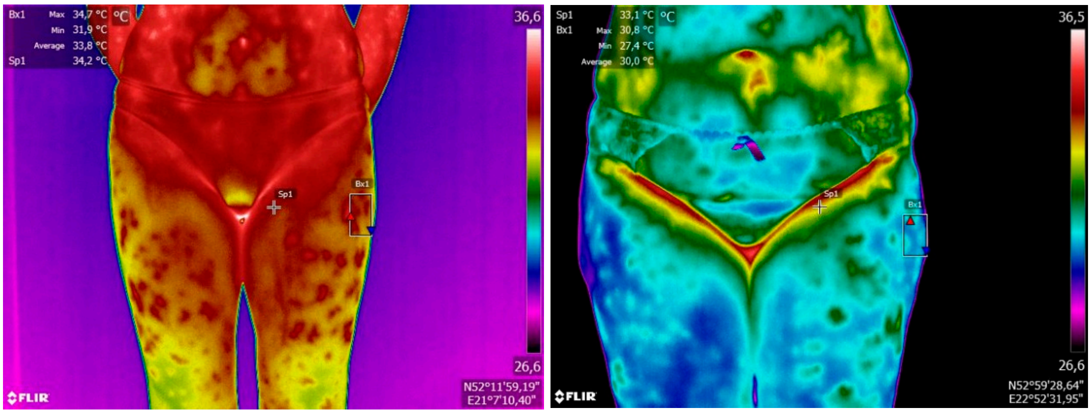

Figure S6. Representative examples of borderline thermographic classification between adjacent cellulite severity grades.

This approach was intended to maximize consistency across raters while minimizing subjective interpretation near category boundaries.
