# Supplementary material for: Inter- and Intra-Rater Reliability of a Standardized Infrared Thermographic Cellulite Severity Classification System: A Multi-Rater Study
Source: J Clin Med. 2026 Sep 20;15(18):7305. doi: 10.3390/jcm15187305 (PMC13608519; doi:10.3390/jcm15187305)
Supplement: Supplementary file 1 [file jcm-15-07305-s001.zip › jcm-4480954-supplementary File S2.pdf]

Supplementary File S2.
Detailed Inter-Rater and Intra-Rater Reliability Analyses

Revised analysis of 78 anonymized FLIR P620 thermographic scans

Analytical scope. The thermographic scan was the unit of analysis. Five raters assigned final ordinal grades in two independently randomized assessment rounds, yielding 780 ratings. The statistical input contained final grades only and contained no participant identifiers or image metadata.

S2.1. Analytical Dataset

Characteristic	Value
**Thermographic scans**	78
**Camera and view**	FLIR P620; anterior
**Acquisition posture**	Upright, relaxed natural standing
**Raters**	5
**Assessment rounds per rater**	2
**Total final grades**	780
**Predefined ordinal scale**	0–IV
**Observed grades**	0–II
**Grade III assignments**	0
**Grade IV assignments**	0

S2.2. Grade Distribution

Grade	Round 1, n (%)	Round 2, n (%)
**0**	258 (66.2%)	285 (73.1%)
**I**	114 (29.2%)	93 (23.8%)
**II**	18 (4.6%)	12 (3.1%)
**III**	0 (0.0%)	0 (0.0%)
**IV**	0 (0.0%)	0 (0.0%)
**Total**	390 (100.0%)	390 (100.0%)

The ratings were concentrated in grades 0 and I. No grade III or IV assignment was observed; therefore, the revised study does not estimate reliability for the upper part of the five-grade scale.

S2.3. Statistical Specification

Primary coefficient. Inter-rater and intra-rater ordinal agreement were quantified using Gwet's AC2 with quadratic weights:

$$AC2 = (P_a - P_e) / (1 - P_e)$$

$$w_{kl} = 1 - [(c_k - c_l) / (cmax - cmin)]^2$$

- Primary AC2 analysis: complete predefined category set 0–IV.
- Additional sensitivity analysis: observed category set 0–II.
- Secondary inter-rater measures: full five-rater exact agreement, full five-rater within-one-grade agreement, nominal Gwet's AC1, Fleiss' kappa, nominal Krippendorff's alpha, and interval-form Krippendorff's alpha.
- Intra-rater analysis: paired round-1 and round-2 ratings for each reader.
- Confidence intervals: non-parametric scan-level bootstrap, 2000 replicates, percentile 95% intervals.
- Inter-rater bootstrap preserved the complete five-rater vector within each sampled scan; intra-rater bootstrap preserved each paired round-1/round-2 rating.
- Random seed: 20260823.

Quadratic weights depend on the category range. A one-grade difference has weight 0.9375 on the full 0–IV scale but weight 0.75 when the weights are recalculated across the observed 0–II range. Both estimates are therefore reported.

S2.4. Inter-Rater Reliability

Statistic	Round 1	95% CI	Round 2	95% CI
**Full five-rater exact agreement**	26.9%	16.7–38.5%	33.3%	23.1–43.6%
**Full five-rater within-one-grade agreement**	89.7%	83.3–96.2%	92.3%	85.9–97.4%
**Gwet AC2, quadratic, full scale 0–IV**	0.949	0.936–0.962	0.963	0.951–0.972
**Gwet AC2, quadratic, observed grades 0–II**	0.786	0.730–0.840	0.845	0.794–0.885
**Gwet AC1, nominal**	0.538	0.462–0.616	0.612	0.536–0.687
**Fleiss kappa, nominal**	0.257	0.130–0.381	0.243	0.114–0.357
**Krippendorff alpha, nominal**	0.259	0.132–0.383	0.245	0.116–0.359
**Krippendorff alpha, interval**	0.320	0.135–0.485	0.327	0.147–0.482

S2.5. AC2 Components

Weighting frame	Round	Weighted observed agreement (P_a)	Expected agreement (P_e)	AC2
**Full scale 0–IV**	Round 1	0.972	0.445	0.949
**Full scale 0–IV**	Round 2	0.977	0.383	0.963
**Observed grades 0–II**	Round 1	0.888	0.475	0.786
**Observed grades 0–II**	Round 2	0.908	0.408	0.845

The higher full-scale AC2 values should be interpreted together with the observed-category sensitivity analysis, exact agreement, within-one-grade agreement, and the nominal coefficients. The full-scale coefficient does not establish reliability for unobserved grades III–IV.

S2.6. Intra-Rater Reliability

Rater	Exact agreement (95% CI)	Within one grade	AC2 full 0–IV (95% CI)	AC2 observed 0–II (95% CI)
**Reader 1**	97.4% (93.6–100.0%)	100.0%	0.997 (0.993–1.000)	0.989 (0.969–1.000)
**Reader 2**	88.5% (80.8–94.9%)	100.0%	0.990 (0.982–0.996)	0.960 (0.927–0.984)
**Reader 3**	79.5% (70.5–88.5%)	100.0%	0.980 (0.970–0.989)	0.919 (0.874–0.957)
**Reader 4**	66.7% (56.4–76.9%)	100.0%	0.955 (0.939–0.970)	0.803 (0.736–0.871)
**Reader 5**	84.6% (75.6–92.3%)	100.0%	0.987 (0.978–0.994)	0.946 (0.910–0.976)
**Mean AC2**	—	—	0.982 (0.976–0.987)	0.923 (0.900–0.944)

All intra-rater disagreements were confined to adjacent grades. Reader 4 showed the lowest exact agreement and the lowest observed-category AC2, indicating heterogeneity in test–retest performance across raters; no unmeasured causal explanation was assigned to this finding.

S2.7. Grade Distribution by Rater

Rater	Round 1: grades 0 / I / II / III / IV	Round 2: grades 0 / I / II / III / IV
**Reader 1**	55 / 21 / 2 / 0 / 0	54 / 21 / 3 / 0 / 0
**Reader 2**	63 / 14 / 1 / 0 / 0	68 / 9 / 1 / 0 / 0
**Reader 3**	55 / 21 / 2 / 0 / 0	64 / 13 / 1 / 0 / 0
**Reader 4**	22 / 44 / 12 / 0 / 0	32 / 40 / 6 / 0 / 0
**Reader 5**	63 / 14 / 1 / 0 / 0	67 / 10 / 1 / 0 / 0

S2.8. Direction of Test–Retest Changes

Rater	One grade lower in round 2	Exact	One grade higher in round 2	Change >1 grade
**Reader 1**	0	76	2	0
**Reader 2**	7	69	2	0
**Reader 3**	13	62	3	0
**Reader 4**	21	52	5	0
**Reader 5**	8	66	4	0
**All raters**	49	325	16	0

Across 390 paired test–retest ratings, 325 were identical, 49 shifted downward by one grade, and 16 shifted upward by one grade. No rater changed a scan by more than one grade.

S2.9. Data and Code Availability

The de-identified scan-level rating matrix and the code reproducing the primary AC2, exact-agreement, within-one-grade, and associated bootstrap analyses are retained by the corresponding authors and are available upon reasonable request. The archived analytical materials contain neutral scan identifiers and final ordinal grades only. They contain no thermographic images, EXIF or GPS metadata, acquisition dates, source filenames, or participant-level identifiers.

S2.10. Measurement-Level Limitation

The analytical dataset contains only the final grade assigned by each rater in each round. Because ROI coordinates, Tmax, Tmin, and ΔT were not archived, the study cannot separately estimate the contributions of ROI placement, temperature extraction, arithmetic calculation, and category assignment to disagreement. The reported coefficients therefore quantify reproducibility of the complete manual scan-to-grade workflow.

S2.11. Interpretation Boundary

- The analyses assess scan-level reproducibility under standardized conditions.
- They do not establish diagnostic accuracy, clinical superiority, or clinical utility.
- The absence of grade III and IV assignments limits inference to the lower severity grades observed.
- Full-scale and observed-category AC2 estimates should be interpreted jointly rather than selecting the larger coefficient in isolation.
- Prospective work should retain ROI coordinates and continuous Tmax, Tmin, and ΔT values to allow component-level reliability analysis.
